# OCULO-AURICULO-VERTEBRAL SPECTRUM ASSOCIATED WITH ABERRANT SUBCLAVIAN ARTERY IN AN INFANT WITH RECURRENT RESPIRATORY DISTRESS

**DOI:** 10.1590/1984-0462/2022/40/2020153

**Published:** 2021-05-26

**Authors:** Amanda Rosa Pereira, Carlos Henrique Paiva Grangeiro, Larissa Cerqueira Pereira, Letícia Lemos Leão, Juliana Cristina Castanheira Guarato

**Affiliations:** a Universidade Federal do Triângulo Mineiro, Uberaba, MG, Brazil.

**Keywords:** Congenital abnormalities, Goldenhar syndrome, Facial asymmetry, Subclavian steal syndrome, Anormalidades congênitas, Síndrome de Goldenhar, Assimetria facial, Estenose da artéria subclávia

## Abstract

**Objective:**

To describe an infant with craniofacial microsomia and recurrent respiratory distress associated with aberrant right subclavian artery in order to review its most frequent congenital anomalies and alert the pediatrician to its rarer and more severe complications.

**Case description:**

This case report involves an 18-month-old male infant, only son of non-consanguineous parents. At birth, the child presented craniofacial dysmorphisms (facial asymmetry, maxillary and mandibular hypoplasia, macrostomia, grade 3 microtia, and accessory preauricular tag) restricted to the right side of the face. Additional tests showed asymmetric hypoplasia of facial structures and thoracic hemivertebrae. No cytogenetic or cytogenomic abnormalities were identified. The patient progressed to several episodes of respiratory distress, stridor, and nausea, even after undergoing gastrostomy and tracheostomy in the neonatal period. Investigation guided by respiratory symptoms identified compression of the esophagus and trachea by an aberrant right subclavian artery. After surgical correction of this anomaly, the infant has not presented respiratory symptoms and remains under multidisciplinary follow-up, seeking rehabilitation.

**Comments:**

Craniofacial microsomia presents a wide phenotypic variability compared to both craniofacial and extracraniofacial malformations. The latter, similarly to the aberrant right subclavian artery, is rarer and associated with morbidity and mortality. The main contribution of this case report was the identification of a rare anomaly, integrating a set of malformations of a relatively common condition, responsible for a very frequent complaint in pediatric care.

## RESUMO

**Objetivo:**

Descrever lactente com microssomia craniofacial e desconforto respiratório recorrente associado à artéria subclávia direita aberrante, com o intuito de revisar as anomalias congênitas mais frequentes e alertar o pediatra sobre suas complicações mais raras e de maior gravidade.

**Descrição do caso:**

Lactente do sexo masculino, 18 meses de idade e filho único de casal não consanguíneo. Ao nascimento, foram observadas dismorfias craniofaciais (assimetria facial, hipoplasia maxilar e mandibular, macrostomia, microtia grau 3 e apêndice na linha trago-oral) restritas ao lado direito da face. Os exames complementares evidenciaram hipoplasia assimétrica da face e hemivértebras torácicas. Não foram identificadas anormalidades citogenéticas ou citogenômicas. O paciente evoluiu com diversos episódios de desconforto respiratório, estridor e náuseas, mesmo tendo realizado gastrostomia e traqueostomia no período neonatal. A investigação direcionada para os sintomas respiratórios identificou a compressão de esôfago e traqueia por uma artéria subclávia direita aberrante. Após a correção cirúrgica dessa anomalia, o lactente não tem apresentado sintomas respiratórios e mantém seguimento multidisciplinar buscando reabilitação.

**Comentários:**

A microssomia craniofacial apresenta grande heterogeneidade fenotípica em relação às malformações tanto craniofaciais como extracraniofaciais. Estas últimas, como a artéria subclávia direita aberrante, são mais raras e associadas à morbimortalidade. A principal contribuição deste relato de caso foi o reconhecimento de uma anomalia rara, integrando um conjunto de malformações de uma condição relativamente comum e sendo responsável por uma queixa muito frequente no atendimento pediátrico.

## INTRODUCTION

The non-random association of anomalies resulting from failures in the development of structures derived from the first and second pharyngeal arches, compromising the formation of craniofacial structures, combined with extracraniofacial malformations, has received different names over time. Currently, no consensus has been reached on whether craniofacial microsomia (CFM), oculo-auriculo-vertebral spectrum (OAVS) (OMIM %164210), hemifacial microsomia, Goldenhar syndrome, and facio-auriculo-vertebral sequence are distinct syndromes or represent a phenotypic spectrum of the first.^1^ ^,^ ^2^

The diagnosis of CFM is clinical and by exclusion. Despite the lack of defined diagnostic criteria, craniofacial malformations seem to be more relevant. These abnormalities include hemifacial microsomia, which results from asymmetric hypoplasia of facial structures and can be recognized by facial asymmetry and mandibular and maxillary hypoplasia. Accessory preauricular tags and abnormalities in the external ear, such as anotia and microtia, are also considered essential.^2^ ^,^ ^3^ ^,^ ^4^

Extracraniofacial manifestations, in turn, are rarer and associated with greater severity. They comprise skeletal changes (mainly in the axial skeleton), malformations of the central nervous system and the urogenital tract, and cardiovascular abnormalities, which may include complex heart diseases and malformations with lower clinical repercussions, such as aortic arch anomalies.^5^

This wide phenotypic heterogeneity is responsible for an incidence variation of 1:3,500-45,000. CFM is the second cause of craniofacial malformations, after orofacial clefts. It has a slight predominance in males (3:2) and greater unilateral impairment, especially on the right side.^6^ ^,^ ^7^

Its etiology is unknown and, in most cases, sporadic. Both dominant and recessive inheritance patterns have been described, suggesting the involvement of genetic variants. More recently, pathogenic variants in the myelin transcription factor 1 (*MYT1*) encoding gene, which participates in the retinoic acid pathway, were detected in patients with this phenotype. Moreover, distinct chromosomal and submicroscopic changes were individually identified in some patients, while 5p deletion and 22q11.2 microdeletion were recurrent.^8^ ^,^ ^9^ ^,^ ^10^ ^,^ ^11^

As in the case of genetic factors, different environmental aspects were associated with CFM. Among them, we can mention those related to pregnancy (gestational diabetes, bleeding, twinning, assisted reproduction techniques, and low birth weight), the use of teratogens (thalidomide, retinoic acid, and vasoactive drugs), and demographic characteristics, including low household income and some ethnic groups, such as Hispanics or Native Americans.^12^

We describe the case of a male infant with the clinical diagnosis of CFM and recurrent respiratory distress associated with aberrant right subclavian artery (ARSA) in order to review the most frequent congenital anomalies related to this syndrome and alert the pediatrician to its rarer and more severe complications.

## CASE REPORT

This case study was developed based on the analysis of medical record data, after the signing of the informed consent form (ICF). The Research Ethics Committee of the Hospital de Clínicas da Universidade Federal do Triângulo Mineiro (HC-UFTM) approved this study.

This report describes an 18-month-old male infant, only son of non-consanguineous parents, with no family history of known genetic syndromes. His mother, aged 38 years during pregnancy, denied using medications, as well as smoking or alcohol consumption. Routine prenatal examinations were normal, except for the detection of polyhydramnios in the third-trimester ultrasound. The patient was born preterm (36 weeks) by cesarean section, weighing 2,695 g (0<z<+1), with 48 cm (0<z<1) height and Apgar score: 8/9. The newborn’s initial examination detected important facial asymmetry (E>D) associated with grade 3 microtia and accessory preauricular tag on the right side. In addition, he showed significant suckling deficits and respiratory distress, receiving a continuous flow of compressed air into the airways through continuous positive airway pressure (CPAP) until he was submitted to tracheostomy and gastrostomy, with 16 days of life. He was discharged with 38 days of life and required supplemental oxygen.

At the age of two months, he was referred to the thoracic surgery outpatient clinic of HC-UFTM for assessment of stridor and increased respiratory distress. He underwent nasopharyngolaryngoscopy, which identified a granuloma just below the tracheostomy opening, and was submitted to resection. In this same period, he was evaluated by the pediatric pulmonology and clinical genetics departments.

**Figure 1 -:**
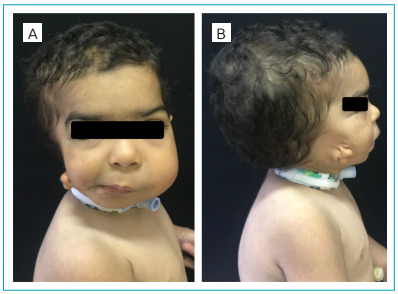
Craniofacial dysmorphisms observed in the infant. (A) Asymmetric hypoplasia of right facial structures, characterized by facial asymmetry (E>D), malar and mandibular hypoplasia, and macrostomia with a cleft in the oral commissure. (B) Abnormalities in the external ear (low-set ear with grade 3 microtia).

The infant’s physical examination revealed underdeveloped right facial structures, associated with thoracic kyphoscoliosis, but without the description of epibulbar dermoids (Figure 1). His craniofacial computed tomography (CT) showed agenesis of various right facial structures, including the upper and lower quadrants of the dental arches, the zygomatic arch, the muscles of mastication, the parotid gland, and the external auditory meatus. No intracranial malformations were found. The urinary tract ultrasound was normal, and the panoramic radiography of the spine identified hemivertebrae at the T11 and T12 level, justifying the thoracic deformity. Evaluation of brainstem auditory evoked potentials (BAEP) confirmed conductive hearing loss in the right ear, and, despite the lack of heart murmur, the patient underwent a transthoracic echocardiogram, which was normal. Lastly, the GTG banding cytogenetic analysis, through lymphocyte culture, and the cytogenomic study, using the CytoSNP-850^®^ high-resolution platform, were normal. The findings of the physical examination, combined with those of the additional tests, led to the diagnosis of CFM.

The infant made several visits to the pediatric emergency department due to nausea, respiratory distress, and stridor. At 5 months of age, he was hospitalized to investigate airway obstruction, and a new nasopharyngolaryngoscopy identified recurrence of granuloma, resulting in significant tracheal stenosis. The patient was again submitted to the removal of the granuloma, followed by dilation and exchange of the longer tracheostomy cannula to avoid new stenoses.

About four months later, the infant presented a new episode of respiratory distress, requiring hospitalization, and was diagnosed with community-acquired pneumonia. On that occasion, he underwent a contrast chest CT, which detected trachea with diffusely thickened walls, a slight caliber reduction below the end of the tracheostomy cannula; change in lung attenuation, with mild opacities; in addition to the presence of ARSA coursing posterior to the esophagus.

At the age of 22 months, the infant underwent surgical ligation and division of the vascular ring, caused by ARSA. No other chest malformation was described during the surgical procedure. The postoperative period was uneventful, and his mother reported that, after the surgical treatment, he presented no other episodes of nausea, or even stridor or respiratory distress. Currently, the patient remains under multidisciplinary follow-up, with good motor progression, and awaits reassessment for craniofacial surgery.

## DISCUSSION

The term CFM is currently used to describe a set of non-random craniofacial and extracraniofacial malformations. Craniofacial anomalies are more easily recognized and appear between the fourth and eighth week of embryonic development, when neural crest cells migrate to future head and neck regions around the stomodeum to form the pharyngeal arches, grooves, pouches, and membranes. In the specific case of CFM, a failure in the development of structures of the first and second pharyngeal arch occurs either due to ischemia or to abnormal migration of neural crest cells. These craniofacial changes are usually unilateral but can also lead to asymmetrical bilateral involvement.^2^ ^,^ ^13^ ^,^ ^14^ The craniofacial anomalies identified in the infant suggested the diagnosis of CFM. His physical examination showed asymmetric hypoplasia of facial structures (E>D), and the cranial CT revealed agenesis of parts of the bone structure and of soft parts on the right side. The patient also presented ear anomalies (grade 3 microtia and atresia of the external auditory meatus), accessory preauricular tag, and macrostomia with a cleft in the oral commissure. This last change results from the failure of fusion of maxillary and mandibular ridges.^13^ We also emphasize that his ophthalmic examination was normal, ruling out ocular abnormalities.

Extracraniofacial malformations, in turn, present a lower frequency, with prevalence ranging from 2-79% of patients with CFM,^15^ and can include skeletal changes, malformations of the central nervous system, cardiovascular abnormalities, and, more rarely, anomalies of the urogenital tract, gastrointestinal tract, and respiratory system.^14^ ^,^ ^15^ Some studies correlate larger craniofacial involvement with a higher frequency of extracraniofacial malformations. Furthermore, since many of these malformations can be oligo/asymptomatic, recognizing them early is essential.^15^ The infant’s initial additional tests identified only vertebral anomalies, since the cranial CT, the echocardiogram, and the urinary tract ultrasound were normal.

Among extracraniofacial malformations, vertebral anomalies are the most common, found in more than half of the patients evaluated (60%). They can include primary defects of formation and segmentation, as well as secondary deformities. The frequency of these anomalies varies with the research methodology, but, in general, vertebral fusion, hemivertebrae, scoliosis, and torticollis are the most frequent. In addition, cervical involvement predominates over the thoracolumbar one. Rib anomalies are rarer and may vary between agenesis, hypoplasia, and fusion.^14^ ^,^ ^15^ ^,^ ^16^ The infant’s radiological evaluation was guided by the kyphoscoliosis observed in his physical examination. This examination was able to identify that the deformity was associated with two thoracic hemivertebrae.

Cardiovascular abnormalities are also an example of extracraniofacial malformations and answer for the higher morbidity and mortality among these patients. Their frequency ranges from 5-58%, and none of them is specific to CFM. Septal, obstructive, and cyanotic defects have been described. Among them, atrioventricular septal defects and tetralogy of Fallot are the most common. Tetralogy of Fallot is an example of conotruncal heart disease associated with failure of neural crest cell migration. In patients with this type of heart disease, the identification of microdeletions involving the q22.1 region of chromosome 22 is quite frequent. Agenesis of the internal carotid artery (ICA) ipsilateral to hemifacial microsomia is the most common vascular malformation.^12^ ^,^ ^17^ ^,^ ^18^ The patient’s echocardiographic investigation was normal, but the research guided by respiratory symptoms identified a common aortic arch anomaly - ARSA. Also, the cytogenomic analysis ruled out the possibility of submicroscopic changes, especially the 22q11.2 microdeletion.

ARSA is the most recurrent branch anomaly of the aortic arch, found in 1 to 1.5% of the general population. ARSA arises as a fourth vessel of the aortic arch, distal to the origin of the left subclavian artery, and crosses the midline between the spine and the trachea. In most cases, this malformation is asymptomatic, but the patient may have symptoms related to the compression of mediastinal structures, such as the esophagus and trachea, as well as aneurysmal dilatation (Kommerell diverticulum) or atherosclerotic changes in this artery. When symptomatic, ARSA is responsible for dysphagia lusoria and, less frequently, for cough, dyspnea, stridor, and airway infections in children, since the trachea is more rigid among adults. Symptoms associated with dilatations of this artery can result in chest pain, ischemia of lower limbs, and/or subclavian steal syndrome.^19^ ^,^ ^20^ ^,^ ^21^ ^,^ ^22^ The symptoms of dysphagia lusoria were not so evident in the infant, since his diet was restricted to gastrostomy; however, his mother reported that, in exceptional oral diet situations, the patient showed increased nausea. The identification and surgical treatment of ARSA allowed not only understanding but also controlling the symptoms of respiratory distress presented by the infant.

The most relevant characteristic of CFM is its heterogeneity. The active search for its major malformations, especially extracraniofacial ones, and associated comorbidities could avoid underdiagnosis, promote a greater understanding of this condition, as well as contribute to preventing its complications. Patients with this condition require multidisciplinary follow-up to identify these possible complications and propose specific treatment. The most significant contribution of this case report was the identification of a rare anomaly, integrating a set of malformations of a relatively common condition, responsible for a very frequent complaint in pediatric care.
